# Diagnostic accuracy of biodex balance system stability indices under single- and dual-task conditions in individuals with chronic ankle instability

**DOI:** 10.1186/s13102-026-01908-1

**Published:** 2026-07-21

**Authors:** Nahid Pirayeh, Aida Shahbazi, Neda Mostafaee, Zahra Kosarian

**Affiliations:** 1 Rehabilitation Research Center, Ahvaz Jundishapur University of Medical Sciences, Ahvaz, Iran; 2 Department of Physiotherapy, School of Rehabilitation Sciences, Ahvaz Jundishapur University of Medical Sciences, Ahvaz, Iran; 3 Iranian Center of Excellence in Physiotherapy, Rehabilitation Research Center, Department of Physiotherapy, School of Rehabilitation Sciences, Iran University of Medical Sciences, Tehran, Iran; 4 Orthopedic Research Center, Mashhad University of Medical Sciences, Mashhad, Iran

**Keywords:** Chronic ankle instability, Balance, Dual-task, Biodex Balance System, Diagnostic accuracy, Rehabilitation

## Abstract

**Background:**

Although balance deficits in chronic ankle instability (CAI) have been well documented, the diagnostic accuracy of instrumented balance measures—particularly under dual-task conditions—for differentiating individuals with CAI from copers remains unclear.

**Objective:**

To examine the discriminative ability and diagnostic accuracy of Biodex Balance System stability indices, with and without a concurrent cognitive task, in differentiating individuals with CAI from copers.

**Methods:**

Seventy participants with a history of lateral ankle sprain were classified into a CAI group (*n* = 35) or a coper group (*n* = 35) based on clinical history and Identification of Functional Ankle Instability (IdFAI) scores. Dynamic balance was assessed using the Biodex Balance System at stability levels 5 and 7. Anterior–posterior (AP), medial–lateral (ML), and overall stability indices were recorded under single-task and dual-task (cognitive counting) conditions. Receiver operating characteristic (ROC) curve analyses were used to determine sensitivity, specificity, area under the curve (AUC), likelihood ratios, predictive values, and optimal cutoff points.

**Results:**

Significant between-group differences were observed for 13 of the 24 assessed stability indices, with medium-to-large effect sizes identified for several ML stability measures under dual-task conditions. The ML stability index at level 5 (eyes open with and without a cognitive task), level 5 with eyes closed and cognitive task, overall stability index at level 5 with eyes open and cognitive task, and ML stability index at level 7 with eyes open and cognitive task yielded moderate AUC values (0.71–0.76). Among these measures, the ML stability index at level 7 with eyes open and cognitive task showed the highest sensitivity (0.74) and lowest negative likelihood ratio (0.40), while the ML stability index at level 5 with eyes open demonstrated the highest specificity (0.75) and positive likelihood ratio (2.44).

**Conclusion:**

Biodex Balance System stability indices, particularly medial–lateral measures obtained under dual-task conditions, demonstrated moderate diagnostic utility for differentiating individuals with CAI from copers. These measures may provide complementary information regarding sensorimotor impairments and may assist clinical assessment and rehabilitation monitoring when used alongside patient history and self-reported instability measures.

**Supplementary Information:**

The online version contains supplementary material available at https://doi.org/10.1186/s13102-026-01908-1.

## Introduction

Lateral ankle sprain is one of the most common musculoskeletal injuries occurring during physical and sporting activities. Although often considered a benign injury, a substantial proportion of individuals experience recurrent sprains or develop chronic ankle instability (CAI), with studies suggesting that up to 70% may develop CAI following an initial sprain [1]. Recurrent ankle sprains are associated with significant long-term consequences, including the development of CAI and post-traumatic ankle osteoarthritis, which has been reported in approximately 68%–78% of cases [2]. A 2021 systematic review reported a prevalence of CAI of approximately 25% in physically active populations, with rates as high as 46% among individuals with a history of a single ankle sprain [3]. According to the International Ankle Consortium, CAI is defined by a history of at least one significant ankle sprain accompanied by recurrent sprains, episodes of “giving way,” or persistent feelings of ankle instability [4]. In contrast, individuals who sustain an ankle sprain but do not develop CAI are referred to as copers. These individuals are able to return to high-level activities, such as jumping and pivoting, without recurrent injury, functional limitations, or persistent instability despite a history of ankle sprain [5]. Clinically, CAI is associated with persistent symptoms including pain, weakness, reduced ankle joint range of motion, repeated periods of immobilization, recurrent injuries, diminished functional capacity, and reduced physical activity levels [6]. Given the strong association between CAI and the later development of ankle osteoarthritis, prevention of CAI following an acute ankle sprain is a critical goal of rehabilitation [4].

Following an ankle sprain, impairments in proprioception, muscle strength, neuromuscular control, and joint mobility are frequently observed, contributing to balance deficits and an increased risk of recurrent injury [7–9]. Numerous studies have examined dynamic balance performance in individuals with CAI compared with healthy controls using the Biodex Balance System. Collectively, these investigations have consistently demonstrated significant impairments in dynamic postural control among individuals with CAI relative to healthy individuals [10–13]. Proposed mechanisms underlying these balance deficits include altered sensory input from joint mechanoreceptors, reduced muscle strength and endurance, mechanical instability of the ankle joint, and restricted ankle range of motion [14].

Balance control is a complex process that arises from the interaction of sensory, motor, and cognitive systems [15]. Emerging evidence suggests that neurocognitive factors play an important role in postural control in individuals with CAI. Beyond local sensorimotor impairments, increased cognitive demands may further compromise movement control and contribute to the risk of recurrent ankle sprains [11, 16, 17]. In everyday activities, balance tasks are rarely performed in isolation and are often accompanied by concurrent cognitive demands [11]. Accordingly, dual-task paradigms—where a balance task is performed simultaneously with a cognitive task—have been increasingly used to assess postural control [11, 12, 16]. Studies by Rahnama et al. (2010) and Shiravi et al. (2012) demonstrated that the addition of a cognitive task during static and dynamic balance assessments significantly reduced postural stability, particularly in the medial–lateral direction, in individuals with functional ankle instability compared with healthy controls [12, 18]. Similarly, Mohammadi et al. (2020) reported greater postural sway during dual-task conditions in individuals with CAI compared with copers and healthy individuals, highlighting the influence of attentional resources on balance control in this population [11].

Although previous research has documented differences in balance performance under single- and dual-task conditions between individuals with CAI and healthy controls, little attention has been given to determining which balance measures possess the greatest diagnostic accuracy for distinguishing individuals with CAI from copers. Identifying measures with strong discriminative ability could enable clinicians to identify individuals at risk for chronic instability, predicting progression toward CAI, and implementing targeted rehabilitation strategies aimed at reducing the prevalence of this condition [19]. Instrumented balance systems, such as the Biodex Balance System, offer quantifiable metrics of postural stability under controlled yet progressively challenging conditions, making them particularly suitable for this purpose. Moreover, incorporating dual-task balance assessments into clinical evaluation may better reflect real-world functional demands, where balance is often challenged concurrently with cognitive tasks.

Therefore, identifying the discriminative ability of balance assessments—particularly determining optimal cutoff values for balance indices—may facilitate earlier detection of CAI-related impairments and inform the development of more effective intervention strategies. Therefore, the aim of this study was to examine the diagnostic accuracy of Biodex Balance System stability indices under single- and dual-task conditions in distinguishing individuals with CAI from coper individuals.

## Methods

### Participants

A total of 70 individuals with a history of ankle sprain participated in this study, including 35 individuals with CAI and 35 copers. Participants were recruited from physiotherapy clinics in Ahvaz, Iran, as well as from staff and students of Ahvaz Jundishapur University of Medical Sciences. Prior to participation, all individuals received a full explanation of the study purpose, procedures, and assessment tools. Written informed consent was obtained from all participants in accordance with the Declaration of Helsinki. The study protocol was approved by the Ethics Committee Committee of Ahvaz Jundishapur University of Medical Sciences (IR.AJUMS.REC.1402.259). Ahvaz Jundishapur University of Medical Sciences, Ahvaz, Iran.

Participants were classified as having CAI if they met all of the following criteria: A history of at least one ankle sprain within the previous 12 months, accompanied by either at least two episodes of ankle “giving way” in the past 6 months, or at least one recurrent ankle sprain within the previous 3–6 months; persistent self-reported symptoms including pain, weakness, or feelings of instability in the involved ankle; failure to return to pre-injury levels of activity; and a score of ≥ 11 on the Identification of Functional Ankle Instability (IdFAI) questionnaire [7, 9, 11]. Participants were classified as coper individuals if they had a history of at least one lateral ankle sprain that resulted in pain, swelling, and interruption of physical activity for at least one day; had experienced no episodes of giving way or recurrent ankle sprain within the previous 12 months; had fully returned to their pre-injury activity levels; and had a score of < 11 on the IdFAI questionnaire [7, 9, 11]. Participants were excluded if they met any of the following criteria: History of ankle joint fracture, History of lower limb surgery within the previous 3 months, History of acute lower limb or head injury within the previous 3 months, Participation in a rehabilitation program for the involved ankle within the previous 3 months [9, 11, 12]. Participants in the coper group were matched to those in the CAI group based on age, sex, body mass index (BMI), and activity level.

### Data collection procedure

After confirming eligibility, demographic and anthropometric data—including age, sex, height, weight, BMI, educational level, and training time—were collected using a standardized form. The IdFAI questionnaire which had 10-item was administered by the examiner to confirm group allocation and assess ankle instability status. Scores < 11 suggest the absence of functional ankle instability, whereas scores ≥ 11 indicate a high likelihood of functional ankle instability [20]. The Persian adaptation of the IdFAI has demonstrated satisfactory psychometric properties, including acceptable validity and reliability, for identifying individuals with functional ankle instability [21]. A physiotherapist, who was blinded to the assignment participants in the groups, implemented the study protocol and recorded the result of tests.

### Balance assessment

Postural control was assessed using the Biodex Balance System (Biodex Medical Systems, Shirley, NY, USA), a widely used instrument for the evaluation of dynamic postural stability in clinical and research settings [10, 12, 22]. The Biodex system quantifies the magnitude of platform oscillations around a zero reference point under dynamic condition and has demonstrated high test–retest reliability for postural stability indices, with intraclass correlation coefficients ranging from 0.89 to 0.93 [10].

Participants performed single-leg stance trials on the involved limb while attempting to maintain the Biodex platform as stable as possible throughout the testing period. During testing, participants stood barefoot with their hands crossed over their chest and were instructed to maintain balance without external support. Balance assessments were conducted under two task conditions: Single-task condition (balance task only), and Dual-task condition (balance task combined with a cognitive task). Each test condition was performed once with eyes open and once with eyes closed. In total, testing was performed under four conditions: eyes open single-task, eyes open dual-task, eyes closed single-task, and eyes closed dual-task. Each trial lasted 30 s and three trials were performed per condition [12]. A short rest interval was provided between trials to minimize fatigue. The order of single-task and dual-task conditions was randomized across participants to reduce potential learning and order effects. The cognitive task consisted of serial subtraction by sevens between two randomly selected numbers (e.g., counting backward from a number between 200 and 300) [18, 23]. Although participants were required to continuously perform the serial subtraction task during the dual-task condition, cognitive task accuracy (e.g., number of correct responses or errors) was not formally recorded or included in the analysis.

The Biodex Balance System platform allows multidirectional tilting around the anterior–posterior (AP) and medial–lateral (ML) axes, with lower stability levels representing greater platform instability. Stability levels 5 and 7 were selected to provide moderate and low platform stability, respectively [12]. Three stability indices were analyzed: the AP stability index, the ML stability index, and the overall stability index, all of which reflect the degree of postural sway in their respective directions [10, 12]. Higher values indicate greater postural sway and poorer balance performance.

### Statistical analysis

Statistical analyses were performed using SPSS software (version 29; SPSS Inc, Chicago, IL). Statistical significance was set at *P* ≤ 0.05. For each test condition, the mean value of three trials was used for analysis. Normality of data distribution was assessed using the Kolmogorov–Smirnov test. Between-group comparisons of demographic variables were conducted using independent t tests for continuous variables and chi-square tests for categorical variables. Group differences in balance outcomes were analyzed using independent t tests, based on the results of K-S test. Cohen’s d effect sizes with corresponding 95% confidence intervals (95% CIs) were calculated to quantify the magnitude and precision of between-group differences. Cohen’s d values of 0.2, 0.5, and 0.8 interpreted as representing small, medium, and large effects, respectively [24]. Receiver operating characteristic (ROC) curve analysis was performed to evaluate the diagnostic accuracy of balance indices. Sensitivity, specificity, area under the curve (AUC), positive and negative likelihood ratios (PLR and NLR), and positive and negative predictive values (PPV and NPV) were calculated for each stability index. The sensitivity of a diagnostic test reflects its capacity to correctly classify individuals who truly have the target condition, whereas specificity indicates how effectively the test identifies individuals who do not have that condition [25–27]. The ROC curve analysis illustrates the relationship between sensitivity (true positive rate) and the false positive rate (1 − specificity) across all possible cutoff values of the balance measures. This approach enables calculation of the area under the curve (AUC) and identification of an optimal threshold for differentiating between study groups [25, 28, 29]. AUC values range from 0 to 1, with values closer to 1 indicating a stronger ability of the test to discriminate between groups, and values near 0 suggesting no discriminative capability. Based on previously established interpretive guidelines for ROC analysis, AUC values of 0.90–1.00 were considered excellent, 0.80–0.89 good, 0.70–0.79 acceptable, 0.60–0.69 poor, and values below 0.60 indicative of insufficient discriminative ability [27, 30, 31]. In the present study, balance indices with AUC values ≥ 0.70 were considered to demonstrate clinically acceptable diagnostic accuracy for differentiating individuals with CAI from copers. The optimal cutoff value was determined using the Youden Index, which identifies the point that maximizes the combined sensitivity and specificity of a test. The Youden Index (J) is calculated as J = sensitivity + specificity − 1 and corresponds to the point closest to the upper-left corner of the ROC curve [28]. To further evaluate the clinical usefulness of the identified cutoff values and the ability of the measures to accurately classify individuals, PLR and NLR, as well as PPV and NPV, were calculated along with their 95% confidence intervals. Likelihood ratios provide robust indicators of diagnostic performance, with higher PLR values supporting confirmation of the condition and lower NLR values supporting its exclusion. Specifically, PLR values greater than 10 and NLR values less than 0.1 are generally considered strong evidence for ruling in or ruling out the condition of interest, respectively [26, 29, 32].

## Results

All participants completed the assessment procedures, and no missing data were observed for the analyzed balance outcomes. Baseline demographic and clinical characteristics of the two groups are presented in Table 1. No statistically significant differences were observed between the CAI and coper groups in terms of age, sex distribution, height, weight, or BMI (*p* > 0.05), indicating appropriate matching between groups. Comparisons of Biodex balance system stability indices under single-task and dual-task conditions are summarized in Table 2. Significant between-group differences (*p* < 0.05) were observed for 13 of the 24 assessed stability indices across single-task and dual-task conditions, with the CAI group generally demonstrating poorer postural control than the coper group. Effect size analysis demonstrated medium-to-large between-group differences for the significant outcomes (absolute Cohen’s |d| = 0.50–1.09), with the largest effects observed for the ML stability indices under dual-task conditions. However, no statistically significant differences were found between groups for the following conditions: AP stability index at stability level 5 with eyes open and eyes closed, ML stability index at stability level 5 with eyes closed, Overall stability index at stability level 5 with eyes open and eyes closed, AP stability index at stability level 7 with eyes open and eyes closed, ML stability index at stability level 7 with eyes closed.

**Table 1:** Demographic information of the study population

Variables	Coper group (*n* = 35) Mean (SD)	CAI group (*n* = 35) Mean (SD)	*P* value
Age (y)	25.9(6.5)	26.8(7.9)	0.60
Height (cm)	170.6(10.4)	167.3(9.7)	0.17
Weight (kg)	67.02(10.9)	69.5(11.6)	0.34
BMI	22.9(2.8)	23.8(2.7)	0.17
Involved foot	21(R), 14(L)	22(R), 13(L)	0.06
Sex	17(M), 18(F)	18(M), 17(F)	0.99
Training time (min)	87.71 (6.45)	84.28 (10.08)	0.09
IdFAI score	8.3(1.7)	18.9(4.2)	0.00

*SD *standard deviation, *CAI *chronic ankle instability, *y *year, *cm *centimeter, *kg *kilogram, *R *right, *L *left, *M *male, *F *female, *min *Minute, *IdFAI *Identification of Functional Ankle Instability

**Table 2:** Descriptive statistics of Biodex Balance System stability indices for each group of coper and chronic ankle instability

Outcome measures	Coper group (*n* = 35) Mean (SD)	CAI group (*n* = 35) Mean (SD)	*P* value	Cohen’s d (95% CI)
AP 7 open	2.46(1.02)	2.94(1.35)	0.11	-0.40 (-0.87, 0.07)
AP 7 open DT	3.01(1.54)	3.85(1.81)	**0.04**	-0.50 (-0.97, -0.01)
AP 7 closed	5.15(1.83)	5.92(1.46)	0.11	-0.46 (-0.93, 0.01)
AP 7 closed DT	5.46(1.99)	6.47(1.97)	0.09	-0.51 (-0.98, -0.02)
ML 7 open	1.68(0.83)	2.32(1.40)	**0.04**	-0.55 (-1.02, -0.06)
ML 7 open DT	1.86(0.74)	2.94(1.41)	**0.00**	-0.95 (-1.44, -0.45)
ML 7 closed	2.96(1.01)	3.57(1.28)	0.10	-0.52 (-1.00, -0.05)
ML 7 closed DT	2.92(1.17)	3.86(1.68)	**0.00**	-0.65 (-1.12, -0.15)
OS 7 open	3.10(1.40)	3.69(1.66)	0.12	-0.38 (-0.85, 0.08)
OS 7 open DT	3.50(1.38)	4.62(1.91)	**0.00**	-0.67 (-1.15, -0.19)
OS 7 closed	5.57(1.85)	6.93(2.10)	**0.00**	-0.69 (-1.16, -0.20)
OS 7 closed DT	5.84(2.19)	6.97(2.21)	**0.03**	-0.51 (-0.98, -0.03)
AP 5 open	2.68(1.65)	3.11(2.13)	0.30	-0.22 (-0.69, 0.24)
AP 5 open DT	3.06(1.57)	4.14(2.38)	**0.02**	-0.54 (-1.01, -0.05)
AP 5 closed	6.32(1.96)	7.26(2.34)	0.16	-0.43 (-0.90, 0.04)
AP 5 closed DT	5.84(2.33)	7.06(2.34)	**0.03**	-0.52 (-0.99, -0.03)
ML 5 open	1.83(0.93)	2.85(1.88)	**0.00**	-0.69 (-1.17, -0.20)
ML 5 open DT	2.07(0.82)	3.68(1.92)	**0.00**	-1.09 (-1.58, -0.58)
ML 5 closed	3.46(1.35)	4.30(2.39)	0.15	-0.43 (-0.90, 0.04)
ML 5 closed DT	3.46(1.52)	5.00(2.02)	**0.00**	-0.86 (-1.34, -0.36)
OS 5 open	3.44(1.91)	3.66(1.79)	0.43	-0.12 (-0.58, 0.34)
OS 5 open DT	3.61(1.63)	5.25(2.30)	**0.00**	-0.82 (-1.30, -0.33)
OS 5 closed	7.01(1.70)	7.74(2.70)	0.53	-0.32 (-0.79, 0.14)
OS 5 closed DT	6.56(2.27)	7.74(2.51)	0.08	-0.49 (-0.96, -0.01)

Biodex stability indices are dimensionless values representing the degree of platform displacement during testing. Higher values indicate greater postural sway and poorer balance performance

*CAI *chronic ankle instability, *SD *standard deviation, *AP *anterior–posterior, *ML *medial–lateral, *OS *overall stability, *DT *dual-task, *CI *confidence interval

Bold values indicate statistically significant differences (*P* < 0.05)

Two-by-two contingency tables for the main diagnostic indices are presented in the Supplementary Material. Receiver operating characteristic (ROC) curve analysis was conducted to evaluate the discriminative ability of the Biodex stability indices between individuals with CAI and copers participants (Tables 3 and 4). The following outcomes demonstrated the highest AUC, ranging from 0.71 to 0.76, indicating moderate discriminative ability: ML stability index at stability level 5 with eyes open, with and without a cognitive task, ML stability index at stability level 5 with eyes closed under dual-task conditions, Overall stability index at stability level 5 with eyes open under dual-task conditions, ML stability index at stability level 7 with eyes open under dual-task conditions. Among all assessed outcomes, the ML stability index at stability level 7 with eyes open and a cognitive task demonstrated the highest sensitivity (0.74; 95% CI: 0.56–0.86), the lowest NLR (0.40; 95% CI: 0.22–0.72), and the highest NPV (0.70; 95% CI: 0.51–0.85). In contrast, the ML stability index at stability level 5 with eyes open yielded the highest specificity (0.75; 95% CI: 0.56–0.86), PLR (2.44; 95% CI: 1.34–4.43), and PPV (0.70; 95% CI: 0.51–0.85).

**Table 3:** Sensitivity, specificity, area under the curve, *P* values, cutoff scores, likelihood ratios, predictive values and 95% confidence intervals for Biodex Balance System stability indices level 7

Outcome measures	Sensitivity (95% CI)	Specificity (95% CI)	AUC (95% CI)	*P* value	Cutoff Score	PLR (95% CI)	NLR (95% CI)	PPV (95% CI)	NPV (95% CI)
AP 7 open	0.54(0.36–0.70)	0.69(0.50–0.82)	0.60(0.47–0.74)	0.11	2.65	1.72(1.00-2.97)	0.66(0.44-1.00)	0.63(0.43–0.79)	0.6(0.43–0.74)
AP 7 open DT	0.54(0.36–0.70)	0.63(0.44–0.78)	0.63(0.50–0.76)	0.04	3.35	1.46(0.87–2.42)	0.72(0.48–1.09)	0.59(0.40–0.75)	0.57(0.40–0.73)
AP 7 closed	0.65(0.47–0.80)	0.55(0.36–0.70)	0.61(0.47–0.74)	0.11	5.45	1.43(0.90–2.27)	0.63(0.38–1.02)	0.58(0.42–0.74)	0.61(0.42–0.77)
AP 7 closed DT	0.65(0.47–0.80)	0.52(0.34–0.68)	0.61(0.48–0.74)	0.09	5.75	1.35(0.86–2.11)	0.66(0.41–1.07)	0.57(0.41–0.72)	0.6(0.40–0.76)
ML 7 open	0.6(0.42–0.75)	0.63(0.44–0.78)	0.63(0.50–0.76)	0.04	1.65	1.61(0.97–2.66)	0.63(0.40–0.99)	0.61(0.43–0.77)	0.61(0.43–0.76)
ML 7 open DT	0.74(0.56–0.86)	0.63(0.44–0.78)	0.73(0.62–0.85)	0.00	2.00	2(1.21–3.28)	0.40(0.22–0.72)	0.66(0.49–0.80)	0.70(0.51–0.85)
ML 7 closed	0.57(0.39–0.73)	0.52(0.34–0.68)	0.61(0.48–0.74)	0.10	3.00	1.17(0.74–1.86)	0.83(0.54–1.27)	0.54(0.37–0.70)	0.54(0.36–0.71)
ML 7 closed DT	0.62(0.44–0.78)	0.63(0.44–0.78)	0.68(0.56–0.81)	0.00	3.15	1.69(1.02–2.79)	0.59(0.37–0.93)	0.62(0.44–0.78)	0.62(0.44–0.78)
OS 7 open	0.62(0.44–0.78)	0.52(0.34–0.68)	0.60(0.47–0.74)	0.12	2.75	1.29(0.82–2.03)	0.72(0.45–1.14)	0.56(0.39–0.71)	0.58(0.39–0.74)
OS 7 open DT	0.62(0.44–0.78)	0.66(0.47–0.80)	0.68(0.55–0.80)	0.00	3.75	1.83(1.09–3.07)	0.56(0.35–0.90)	0.64(0.46–0.79)	0.63(0.46–0.78)
OS 7 closed	0.74(0.56–0.86)	0.66(0.47–0.80)	0.68(0.56–0.81)	0.00	6.00	2.16(1.29–3.62)	0.39(0.21–0.69)	0.68(0.51–0.81)	0.71(0.53–0.85)
OS 7 closed DT	0.71(0.53–0.84)	0.66(0.47–0.80)	0.64(0.51–0.77)	0.03	6.00	2.08(1.24–3.48)	0.43(0.25–0.74)	0.67(0.50–0.81)	0.69(0.51–0.83)

*CI *confidence interval, *DT *dual-task, *AP *Anterior–posterior, *ML *Medial–lateral, *OS *Overall stability, *AUC *Area under the curve, *PLR *Positive likelihood ratio, *NLR *Negative likelihood ratio, *PPV *Positive predictive value, *NPV *Negative predictive value

**Table 4:** Sensitivity, specificity, area under the curve, *P* values, cutoff scores, likelihood ratios, predictive values and 95% confidence intervals for Biodex Balance System stability indices level 5

Outcome measures	Sensitivity (95% CI)	Specificity (95% CI)	Area under the curve (95% CI)	*P* value	Cutoff Score^*^	PLR (95% CI)	NLR (95% CI)	PPV (95% CI)	NPV (95% CI)
AP 5 open	0.57(0.39–0.73)	0.55(0.36–0.70)	0.57(0.43–0.70)	0.30	2.25	1.25(0.78–1.99)	0.78(0.51–1.20)	0.55(0.38–0.71)	0.55(0.38–0.72)
AP 5 open DT	0.6(0.42–0.75)	0.69(0.50–0.82)	0.65(0.53–0.78)	0.02	3.15	1.90(1.11–3.27)	0.58(0.37–0.91)	0.65(0.46–0.80)	0.63(0.45–0.77)
AP 5 closed	0.57(0.39–0.73)	0.60(0.42–0.75)	0.59(0.46–0.73)	0.16	6.35	1.42(0.87–2.33)	0.71(0.46–1.09)	0.58(0.40–0.74)	0.58(0.40–0.74)
AP 5 closed DT	0.62(0.44–0.78)	0.60(0.42–0.75)	0.65(0.52–0.78)	0.03	6.25	1.57(0.96–2.55)	0.61(0.38–0.98)	0.61(0.43–0.76)	0.61(0.43–0.77)
ML 5 open	0.62(0.44–0.78)	0.75(0.56–0.86)	0.72(0.60–0.84)	0.00	2.00	2.44(1.34–4.43)	0.5(0.31–0.79)	0.70(0.51–0.85)	0.66(0.49–0.80)
ML 5 open DT	0.68(0.50–0.82)	0.72(0.53–0.84)	0.76(0.65–0.87)	0.00	2.35	2.40(1.36–4.21)	0.44(0.26–0.73)	0.70(0.52–0.84)	0.69(0.51–0.83)
ML 5 closed	0.60(0.42–0.75)	0.55(0.36–0.70)	0.59(0.46–0.73)	0.15	3.25	1.31(0.82–2.08)	0.73(0.47–1.14)	0.56(0.39–0.72)	0.57(0.39–0.74)
ML 5 closed DT	0.68(0.50–0.82)	0.60(0.42–0.75)	0.71(0.59–0.83)	0.00	4.00	1.71(1.05–2.77)	0.52(0.31–0.87)	0.63(0.45–0.77)	0.65(0.46–0.80)
OS 5 open	0.65(0.47–0.80)	0.46(0.29–0.63)	0.55(0.41–0.69)	0.43	2.65	1.21(0.78–1.86)	0.75(0.46–1.21)	0.54(0.38–0.69)	0.57(0.37–0.74)
OS 5 open DT	0.65(0.47–0.80)	0.72(0.53–0.84)	0.71(0.58–0.83)	0.00	4.25	2.3(1.30–4.04)	0.48(0.29–0.78)	0.69(0.51–0.83)	0.67(0.50–0.81)
OS 5 closed	0.54(0.36–0.70)	0.46(0.29–0.63)	0.54(0.40–0.68)	0.53	6.50	1(0.63–1.56)	1(0.66–1.50)	0.5(0.33–0.66)	0.5(0.32–0.67)
OS 5 closed DT	0.65(0.47–0.47)	0.55(0.36–0.70)	0.61(0.48–0.75)	0.08	6.90	1.43(0.90–2.27)	0.63(0.38–1.02)	0.58(0.42–0.74)	0.61(0.42–0.77)

*CI *confidence interval, *DT *dual-task, *AP *Anterior–posterior, *ML *Medial–lateral, *OS *Overall stability, *AUC *Area under the curve, *PLR *Positive likelihood ratio, *NLR *Negative likelihood ratio, *PPV *Positive predictive value, *NPV *Negative predictive value

*Statistically significant difference (*P* < 0.05)

## Discussion

The present study investigated the discriminative ability of Biodex Balance System stability indices to differentiate individuals with CAI from copers. Our findings indicate that several stability indices demonstrated moderate diagnostic accuracy, with AUC values ranging from 0.71 to 0.76. Specifically, the ML stability index at level 5 with eyes open, both with and without a concurrent cognitive task, the ML stability index at level 5 with eyes closed and a cognitive task, the overall stability index at level 5 with eyes open and a cognitive task, and the ML stability index at level 7 with eyes open and a cognitive task showed the highest discriminative performance. Although these findings suggest that selected Biodex Balance System conditions may help distinguish individuals with CAI from coper individuals, the magnitude of the AUC values indicates only moderate, rather than strong, diagnostic utility. From a clinical perspective, the likelihood ratios and predictive values observed in the present study suggest moderate diagnostic usefulness of the Biodex Balance System indices. The highest PLR observed was 2.44, indicating that individuals with a positive test result were approximately 2.44 times more likely to have CAI than copers. Although this finding suggests some ability to support the presence of CAI, PLR values greater than 5 or 10 are generally considered necessary to provide strong confirmatory evidence in clinical practice [33–35]. Similarly, the lowest NLR observed was 0.40, indicating that a negative test result moderately reduced the probability of CAI; however, NLR values below 0.1 are typically considered strong evidence for ruling out a condition [33–35]. The PPV and NPV findings also demonstrated moderate classification performance. For example, the highest PPV value of 0.70 suggests that approximately 70% of participants with a positive test result truly had CAI, whereas the highest NPV value of 0.70 indicates that approximately 70% of participants with a negative result were correctly classified as copers. However, these predictive values should be interpreted with caution. Because the study design included an equal number of participants in the CAI and coper groups, the estimated prevalence of CAI in the sample was approximately 50%, which does not reflect real-world clinical or athletic populations. Given that PPV and NPV are strongly influenced by disease prevalence, these values may differ substantially in populations with lower or higher CAI prevalence [32, 35]. Therefore, likelihood ratios and AUC values may provide more generalizable indicators of diagnostic performance across different clinical settings. Collectively, these findings indicate that Biodex stability indices, particularly ML indices, may contribute to clinical assessment when used alongside history, patient-reported instability, and functional examination, rather than serving as stand-alone diagnostic tools.

Across all tested conditions, stability indices in the ML direction demonstrated greater discriminative ability than those in other directions. This finding is biomechanically and clinically meaningful. Individuals with CAI are particularly sensitive to excessive inversion and supination movements, which place the talocrural joint in a mechanically vulnerable position characterized by reduced bony congruence and stability [2]. Excessive inversion increases the risk of recurrent ankle sprains, a hallmark of CAI [36]. In addition, in lateral ankle sprain injuries, the anterior talofibular and calcaneofibular ligaments are most frequently damaged, leading to impaired joint mechanoreceptor function, proprioceptive deficits, and altered neuromuscular control [2, 37]. Repeated exposure to destabilizing loads further exacerbates deficits in ML stability [13]. Furthermore, structural adaptations observed in individuals with CAI—such as inferior and anterior displacement of the distal fibula—result in increased laxity of the anterior talofibular ligament at rest and delayed tensioning during medial–lateral movements, thereby reducing its protective role [38].

In addition to ligamentous alterations, neuromuscular impairments contribute substantially to instability. Reductions in the strength and coordination of invertor and evertor muscle groups, which provide dynamic control in the ML direction, have been consistently reported in individuals with CAI [39]. Under normal conditions, rapid activation of the peroneal muscles serves as a protective mechanism against excessive inversion. However, delayed peroneal muscle reaction times in individuals with CAI further compromise ML stability [40]. Moreover, alterations in subtalar joint biomechanics—specifically, lateral displacement of the subtalar joint axis—increase the likelihood of medial center-of-pressure shifts during weight bearing. This results in greater supination torque, which must be counteracted by the peroneal muscles and lateral ankle ligaments. Given the compromised function of these structures in CAI, balance control in the ML direction is disproportionately affected [2, 36]. Collectively, these biomechanical and neuromuscular factors explain why ML stability indices were the most effective discriminators between groups. These findings align with recent evidence, including the study by Kim et al. (2025), which also reported greater balance impairment in the ML direction in individuals with CAI [41].

Another important finding of this study is that dual-task conditions, in which a cognitive task was added to the balance task, produced the greatest discriminative power between groups. This supports growing evidence that cognitive loading exacerbates balance deficits in individuals with musculoskeletal disorders, including CAI [11, 12, 26]. Despite rehabilitation, individuals with CAI often report persistent difficulty performing daily activities in complex, real-world environments, where cognitive demands are higher than in controlled clinical settings [42]. Given the limited capacity of attentional resources, increased cognitive load interferes with motor control, particularly in individuals with impaired proprioception and sensorimotor integration [11]. When a secondary cognitive task is imposed, individuals with CAI appear less able to employ compensatory strategies due to deficits in joint mechanoreceptors and proprioceptive feedback, resulting in greater deterioration of balance performance [12, 18]. In contrast, coper individuals demonstrate greater adaptability of the sensorimotor system and more effective allocation of attentional resources, allowing them to maintain postural stability under dual-task conditions.

The present findings also indicate that stability level 5 of the Biodex Balance System exhibited the greatest ability to differentiate between individuals with CAI and copers. As platform instability increases, reliance on proprioceptive input and precise neuromuscular control becomes more critical [4]. Because these systems are compromised in CAI, balance impairments become more pronounced at higher instability levels. Our results suggest that level 5 provides an optimal challenge that amplifies group differences without overwhelming postural control in coper individuals. This observation is consistent with previous findings, including those of Rahnama et al. (2010), who similarly reported a greater difference between individuals with CAI and healthy individuals in the stability level of the 5 Biodex balance system [12].

### Clinical implications

The present findings may have important implications for rehabilitation in individuals with CAI. The greater impairments observed in medial–lateral stability, particularly under dual-task conditions, suggest that rehabilitation programs should incorporate exercises targeting dynamic postural control in the medial–lateral direction while simultaneously challenging attentional and cognitive processing demands. Traditional rehabilitation approaches often emphasize isolated balance activities under relatively simple conditions; however, the current results indicate that balance deficits in individuals with CAI become more pronounced when cognitive load is increased, which may better reflect the demands encountered during sport and daily activities. Accordingly, rehabilitation programs for individuals with CAI may benefit from integrating dual-task balance training, such as single-leg stance exercises on unstable surfaces combined with cognitive tasks, as well as sport-specific activities that challenge dynamic postural control and attentional processing simultaneously.

### Limitations and future directions

Several limitations should be considered when interpreting the findings of this study. First, the cross-sectional design limits causal inferences regarding the relationship between balance impairments and the development or progression of CAI. Longitudinal studies are needed to determine whether the identified balance indices can predict future instability or recurrent injury. Second, the sample size, although adequate for diagnostic accuracy analysis, may limit generalizability to broader populations, such as elite athletes, older adults, or individuals with bilateral ankle instability. Third, only one type of cognitive task was used; different cognitive loads or task complexities may influence balance performance differently and should be explored in future research. Furthermore, the accuracy of the cognitive task was not formally measured; therefore, it was not possible to determine whether participants prioritized the cognitive or balance task during dual-task testing or whether cognitive task performance differed between groups. Another limitation of this study is that classification of participants with CAI and copers was based primarily on injury history and the IdFAI questionnaire without inclusion of a standardized clinical examination. Although the IdFAI is a validated and widely used instrument for identifying functional ankle instability, the absence of objective clinical assessment may have increased the risk of diagnostic misclassification or bias. In addition, some aspects of the inclusion criteria did not fully align with the recommendations of the International Ankle Consortium. The minor differences in inclusion criteria may limit comparability with studies strictly adhering to Consortium criteria. Future studies should incorporate comprehensive clinical examination criteria alongside patient-reported outcome measures to strengthen participant classification. Furthermore, cut-off values were derived from the same dataset used to construct the ROC curves, which may introduce overfitting and limit the generalizability of these thresholds to other populations. Future studies using independent validation samples are recommended to confirm the stability and external validity of these cutoff values. Another limitation of this study is the large number of statistical comparisons increases the risk of Type I error. Although no formal correction (e.g., Bonferroni or false discovery rate) was applied due to the exploratory nature of the study, the findings should be interpreted cautiously and require confirmation in future studies.

## Conclusion

Although CAI and coper status are commonly classified using injury history and validated self-report questionnaires, objective balance assessment may provide clinically meaningful information beyond subjective reporting alone. Individuals with similar ankle sprain histories may demonstrate different levels of sensorimotor adaptation and postural control. Therefore, instrumented balance testing may help clinicians identify residual functional impairments that are not fully captured through questionnaires or injury history. Moreover, dual-task balance assessments may better reflect the cognitive and motor demands encountered during sport and daily activities. Accordingly, the findings of the present study support the use of Biodex Balance System indices as complementary functional assessment tools within a comprehensive clinical evaluation framework, rather than as stand-alone diagnostic methods for classifying CAI.

## Supplementary Information

Supplementary Material 1.

## Data Availability

The datasets used in this study are available upon request from the corresponding author. Access to the data is subject to any applicable ethical and legal approvals.
