# Supplementary material for: Diagnostic accuracy of biodex balance system stability indices under single- and dual-task conditions in individuals with chronic ankle instability
Source: BMC Sports Sci Med Rehabil. 2026 Jul 21;18:496. doi: 10.1186/s13102-026-01908-1 (PMC13673776; doi:10.1186/s13102-026-01908-1)
Supplement: Supplementary file 1 [file 13102_2026_1908_MOESM1_ESM.docx]

**Supplementary Table S1. Contingency tables (2×2) for the main Biodex Balance System diagnostic indices**

| Variable | TP | FP | FN | TN |
| --- | --- | --- | --- | --- |
| ML 7 open DT | 26 | 13 | 9 | 22 |
| ML 5 open | 22 | 9 | 13 | 26 |
| ML 5 open DT | 24 | 10 | 11 | 25 |
| ML 5 closed DT | 24 | 14 | 11 | 21 |
| OS 5 open DT | 23 | 10 | 12 | 25 |

TP, true positive; FP, false positive; FN, false negative; TN, true negative; ML, medial–lateral; OS, overall stability; DT, dual-task.

**Notes for Interpretation**

- TP: Participants with chronic ankle instability (CAI) correctly identified as positive.
- FP: Coper participants incorrectly classified as CAI.
- FN: Participants with CAI incorrectly classified as copers.
- TN: Coper participants correctly identified as negative.

The values were derived using the optimal cutoff points identified from ROC curve analysis.
